# Scavenging Ducks and Transmission of Highly Pathogenic Avian Influenza, Java, Indonesia

**DOI:** 10.3201/eid1608.091540

**Published:** 2010-08

**Authors:** Joerg Henning, Hendra Wibawa, John Morton, Tri Bhakti Usman, Akhmad Junaidi, Joanne Meers

**Affiliations:** Author affiliations: The University of Queensland, Brisbane, Queensland, Australia (J. Henning, J. Morton, J. Meers);; Australian Animal Health Laboratory, Geelong, Victoria, Australia (H. Wibawa);; Disease Investigation Centre, Wates, Indonesia (T.B. Usman, A. Junaidi)

**Keywords:** Avian influenza, ducks, chickens, Indonesia, HPAI, viruses, transmission, research

## Abstract

In Java, Indonesia, during March 2007–March 2008, 96 farms with scavenging ducks that were not vaccinated against highly pathogenic avian influenza (HPAI) were monitored bimonthly. Bird-level (prevalence among individual birds) H5 seroprevalence was 2.6% for ducks and 0.5% for chickens in contact with ducks. At least 1 seropositive bird was detected during 19.5% and 2.0% of duck- and chicken-flock visits, respectively. Duck flocks were 12.4× more likely than chicken flocks to have seropositive birds. During 21.4% of farm visits, ≥1 sampled duck was H5 seropositive when all sampled in-contact chickens were seronegative. Subtype H5 virus was detected during 2.5% of duck-flock visits and 1.5% of chicken-flock visits. When deaths from HPAI infection occurred, H5 virus shedding occurred in apparently healthy birds on 68.8% of farms. Of 180 poultry deaths investigated, 43.9% were attributed to H5 virus. These longitudinal study results indicate that ducks are a source of infection for chickens and, potentially, for humans.

## Abstract 2

These ducks may be a source of infection for chickens and humans.

Since 1997, when highly pathogenic avian influenza (HPAI) subtype H5N1 outbreaks occurred in poultry in Hong Kong, People’s Republic of China (*1**–**2*), the virus has caused epidemics in Asia, Europe, and Africa (*3*). In Indonesia, the first HPAI (H5N1) virus infections in poultry were officially announced in early 2004 (*4*); human cases have been reported since mid-2005 (*5*). Although extensive HPAI control efforts helped reduce the frequency of outbreaks in poultry (*6*), by 2009, subtype H5N1 virus had been detected in 31 of Indonesia’s 33 provinces (*7*). In 2009, Indonesia had the highest incidence worldwide of human infections and deaths (*8*).

Waterfowl are the natural reservoir of avian influenza viruses (*9*), and experimental research indicates that ducks may play a role in the maintenance of HPAI (H5N1) viruses. Infected ducks may exhibit no clinical signs yet can excrete high concentrations of virus that are pathogenic to other poultry species (*10**–**13*). Possible risk factors for HPAI spread in Indonesia include duck movements, contacts between ducks and other poultry and animal species, poor poultry husbandry, inadequate handling of sick and dead ducks by flock owners, and poor awareness of control strategies among poultry farmers (*14*). However, no analytical study assessing risk factors for HPAI infection has been conducted in Indonesia.

In 2005, Indonesia’s duck population was ≈34.3 million, of which 40% were on the island of Java, mainly on small-holder farms, i.e., backyard and small commercial farms (*14*). As in many other Asian countries, domestic ducks on small-holder farms in Indonesia are allowed to scavenge freely during the day around houses, in the villages, or in rice paddies; duck owners supply little or no feed (*15*). To assess the hypothesis that ducks contribute to the maintenance and transmission of avian influenza (H5N1) viruses, we conducted a longitudinal investigation describing temporal patterns of antibodies against HPAI (H5) and virus prevalence in unvaccinated scavenging ducks and chickens that have contact with these ducks (in-contact chickens) in Java, Indonesia.

## Materials and Methods

### Study Design

**Figure 1:**
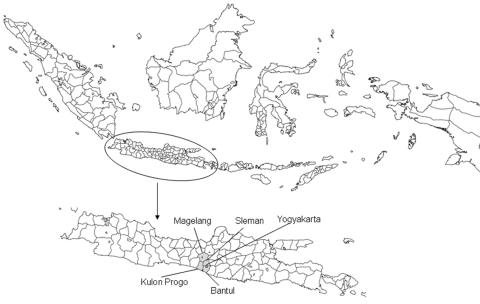
Location of districts in Central Java, Indonesia, where ducks and in-contact chickens were monitored bimonthly for avian influenza (H5) during March 2007–March 2008.

Ducks and in-contact chickens on 96 small-holder duck farms in 4 districts of Central Java were monitored once every 2 months over 13 months. Four districts were selected (Sleman, Magelang, Bantul, and Kulon Progo) because of their high abundance of duck farms and proximity to the Disease Investigation Center (DIC) in Wates, where field investigators were based and diagnostic work was conducted (Figure 1). Sample size calculations were based on DIC surveillance data collected in Central Java in 2006; 13 (4.7%) of 278 cloacal swabs from ducks were positive for H5 viral RNA on real-time reverse transcription–PCR (RT-PCR). On the basis of an expected true bird-level virus prevalence of 5%, a precision of the estimate of ±1.5 % and a 95% confidence interval (CI), a total of 811 ducks had to be sampled (*16*). We enrolled 96 duck farms in the study and sampled a total of 960 ducks (10 ducks per farm) and 480 in-contact chickens (5 chickens per farm) during each of 7 visits over 13 months (initial visit plus 6 bimonthly visits).

We used a multistage sampling strategy with stratification by district and a 3-level sampling process that involved villages, duck farms, and birds. A sampling frame was prepared by agriculture extension officers who listed all villages in the selected districts, including the total number of duck farms within each village. We selected 4 villages within each district using probability proportional to size sampling. Field veterinarians then prepared a second sampling frame containing the names of all duck farmers within the 16 villages selected and the number of ducks kept by each farmer. From this sampling frame, 6 duck farms per village were selected by using simple random sampling. Farms with <10 ducks were excluded (we wanted to sample 10 ducks per farm) as were farms with >700 ducks (which we considered to be large commercial farms). Random numbers for village and duck farm selection procedures were produced in STATA version 10.0 (Stata Corporation, College Station, TX, USA).

### Data Collection and Diagnostic Tests

Four veterinarians from the DIC were trained in the use of data collection tools and interviewing techniques. Field visits were conducted once every 2 months from March 2007 through March 2008; duck owners were interviewed and swab and blood samples from birds were obtained during each visit. On the first visit, birds were selected for the study. The duck owner enclosed all ducks in a pen and selected the first 10 ducks that could be caught. If available, 5 chickens kept on the same farm were also selected in the same manner. Wing tags or leg bands were attached to each selected duck and chicken. Blood samples were collected from the wing vein of each bird, and an oropharyngeal swab and a cloacal swab were collected from each bird and placed into a single tube containing virus transport media (Universal Viral Transport 3mL; Becton Dickinson, Franklin Lakes, NJ, USA). Duck owners confirmed that none of the ducks and chickens sampled had been vaccinated against HPAI before the study and that none were vaccinated during the study.

Serum samples were tested for antibodies to avian influenza (H5) by using the hemagglutination inhibition (HI) test according to methods recommended by the World Organisation for Animal Health (OIE) (*17*). Antigen and control antiserum were supplied from Pusat Vetenerinaria Farma (Surabaya, Indonesia). The antigen was derived from an HPAI (H5N1) chicken isolate obtained in 2004 in Indonesia (A/chicken/Pare/East Java/2004). This antigen is commonly used for HI tests to detect antibodies to avian influenza (H5N1) at all veterinary diagnostic laboratories in Indonesia. A titer >2^4^ against 4 hemagglutinating units of antigen was considered positive (*17*). In accordance with the Australian Animal Health Laboratory protocol (*18**–**19*), RT-PCR was used to test the combined oropharyngeal and cloacal swabs of individual birds in pools of 5 for subtype H5 virus RNA. Sequencing was conducted on the H5 RT-PCR–positive samples to confirm the HPAI multiple basic amino acid motif at the cleavage site of the hemagglutinin gene and to determine whether the neuraminidase gene of the isolate belonged to the N1 subtype.

### Investigations of Bird Deaths

Duck farmers involved in the study were asked to immediately report sickness or deaths of birds to the DIC. Compensation was paid to duck farmers to encourage reporting. Upon notification, veterinarians conducted an outbreak investigation at the reported farm by using a predesigned questionnaire. Clinical signs were recorded, and carcasses were collected for postmortem examination. Blood and swab samples from clinically normal birds from the same farm were obtained on the day of the investigation. Blood samples were tested for antibodies to avian influenza (H5) as already discussed; swab samples from carcasses (combining lung, heart, liver, spleen, pancreas, and intestinal tissues) and from live birds were processed by virus isolation in embryonated eggs. Two passages of virus isolation were conducted, and allantoic fluid was tested for H5 antigen of avian influenza by using the HI test. An HPAI outbreak was defined as >1 bird dying within a few days of each other from HPAI (i.e., positive by subtype H5 virus isolation or RT-PCR).

### Data Analyses

For both ducks and in-contact chickens, bird-level seroprevalence (proportion of study birds with antibodies to avian influenza [H5]) and flock-level seroprevalence (in which at least 1 study bird had antibodies) were calculated for each of the 7 sampling periods and pooled across the entire study period. Virus prevalence was calculated only at flock level (proportion of flock visits in which at least 1 pool of swab samples from the farm was positive for H5 RNA) for the entire study period. We accounted for the multistage sampling strategy in the data analyses by using survey commands in STATA version 10.0 (Stata Corporation); districts were treated as strata; villages were specified as primary, and farms as secondary, sampling units. For bimonthly bird-level prevalences, and for bird- and flock-level prevalences over the entire study period, sampling weights were the inverse of the product of the proportion of villages in the district that were sampled and the proportion of duck farms in the village that were sampled (*20*). The finite population correction factor for primary sampling units was the total number of villages in the district. Finite population correction accounted for reduction in variance associated with sampling without replacement (*21*). For bird-level seroprevalence calculations over the entire study period, we accounted for repeated measurements within the same birds by specifying the individual bird as the third level of sampling and incorporating the number of duck farms per village as the finite population correction factor for secondary sampling units. For the bimonthly flock-level seroprevalence, only primary sampling units with their finite population correction factor were specified in the analyses. Sampling weights for bimonthly flock-level seroprevalence were the inverse of the proportion of villages in the sampled district.

We used logistic regression models accounting for 3 levels of clustering (birds within farms within villages) to compare the odds of birds having titers positive for avian influenza (H5) between ducks and in-contact chickens. For flock-level comparisons, logistic regression models accounting for 2 levels of clustering (farms within villages) were used to compare the odds of flocks having at least 1 bird with antibodies to avian influenza (H5) between duck and in-contact chicken flocks, and for duck flocks between sampling months. Logistic regression accounting for 2 levels of clustering was also used to evaluate whether the odds of duck (or chicken) flocks being seropositive were independent of the results of the other species at the same farm and sampling. All logistic regression models also accounted for sampling weights and incorporated finite population correction. Adjusted Wald tests were used to assess the overall effect of sampling month. After fitting the logistic regression models taking the survey sampling design into account, we applied the F-adjusted mean residual goodness-of-fit test (*22*).

## Results

From March 2007 through March 2008, a total of 8,993 serum and swab samples were collected from 6,705 clinically healthy ducks and 2,288 chickens during 670 farm visits (at 2 farm visits, all birds had been sold). During ≈80% of farm visits, chickens were also present. Flock sizes for ducks and chickens averaged 53.7 and 8.5, respectively. Of all combined oropharyngeal and cloacal swab sets from individual birds, 8,900 were analyzed in pools of 5 by RT-PCR, and all serum samples were tested for antibodies to subtype H5 virus. In addition, during outbreak investigations, 174 sets of swabs from dead birds and 136 from apparently healthy live birds were collected from the outbreak farms.

### Prevalence of Antibodies to Avian Influenza (H5)

Bird-level seroprevalences of subtype H5 antibody titers >2^4^ in clinically healthy birds for all bird samplings pooled over the entire study period were 2.6% (95% CI 1.8–3.5) for ducks and 0.5% (95% CI 0.0–0.9) for in-contact chickens. The odds of ducks being positive for avian influenza (H5) were 5.5× (95% CI 2.1–14.4) higher than for in-contact chickens. Flock-level seroprevalence of antibodies to avian influenza (H5) was 19.5% (95% CI 14.3–24.6) for ducks and 2.0% (95% CI 0.1–3.9) for in-contact chickens. The odds of duck flocks being seropositive were 12.4× (95% CI 3.9–40.1) higher than those for chicken flocks.

**Figure 2:**
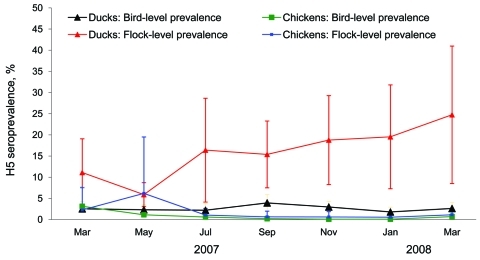
Bird- and flock-level seroprevalences of avian influenza (H5) in ducks and in-contact chickens monitored for infection, Central Java, Indonesia, March 2007–March 2008. Error bars indicate point-wise 95% confidence intervals. Flock-level seroprevalences are proportions of flocks where at least 1 bird had an antibody titer >2^4^ to H5 virus. Estimates are adjusted for the survey structure.

Figure 2 shows the H5 bird- and flock-level seroprevalences for ducks and in-contact chickens from the beginning of March 2007 through the end of March 2008. Duck flock–level prevalence varied over time from 5.9% to 24.7%. The odds of a duck flock being seropositive differed significantly by month (p = 0.05); odds were higher in July 2007 (odds ratio [OR] = 3.1; 95% CI 1.1–9.0), September 2007 (OR = 2.9; 95% CI 1.4–6.3), November 2007 (OR = 3.7; 95% CI 1.7–8.1) 2007, January 2008 (OR = 3.9; 95% CI 1.5–10.0), and March 2008 (OR = 5.3; 95% CI 1.9–14.7), relative to May 2007. Chicken flock-level seroprevalences remained <6.2% throughout the study.

At 21.4% of 501 farm visits, >1 study duck was seropositive for influenza (H5) when during the same farm visits, all in-contact study chickens on these farms were seronegative (Table). Conversely, at only 1.4% of farm visits was >1 study chicken seropositive for avian influenza (H5), while all study ducks on the farm were seronegative. At flock level, seropositivity of ducks was not associated with seropositivity of chickens on the same farm (OR = 3.9, 95% CI 0.4–43.0). The goodness-of-fit statistics calculated after fitting the survey design–adjusted logistic regression models provided no evidence of lack of fit of any of the models (p>0.05).

**Table:** Antibodies to avian influenza (H5) in ducks and in-contact chickens monitored on the same farms during 501 farm visits, Central Java, Indonesia, March 2007–March 2008

H5 serologic status of ducks*	H5 serologic status of in-contact chickens†	No. (%) farm visits
Positive	Positive	3 (0.6)
Positive	Negative	107 (21.4)
Negative	Positive	7 (1.4)
Negative	Negative	384 (76.6)

*Based on results from 10 ducks at each farm visit. †Based on results from 5 in-contact chickens at each farm visit.

### HPAI (H5) Virus Prevalence in Clinically Healthy Birds

Birds on 25 (26%) of the 96 monitored farms tested positive for avian influenza (H5) virus RNA; on 20 farms, birds tested positive on 1 sampling occasion and, on 5 farms, on 2 different sampling occasions. On these 25 farms, 30 flocks (22 duck and 8 chicken flocks) tested positive for subtype H5 virus RNA. On 3 farms, both duck and chicken flocks tested positive for subtype H5 virus RNA at the same visit (6 flocks); otherwise, only 1 flock (either ducks or chickens) was positive for subtype H5 virus RNA at any 1 visit (19 duck and 5 chicken flocks). The flock prevalence of subtype H5 virus RNA (proportion of flock-visits during which at least 1 study bird was positive) in clinically healthy birds for all flock samplings pooled over the entire study period was 2.5% (95% CI 0.9–4.1) for ducks and 1.5% (95% CI 0.4–2.7) for chickens.

### HPAI Outbreaks

**Figure 3:**
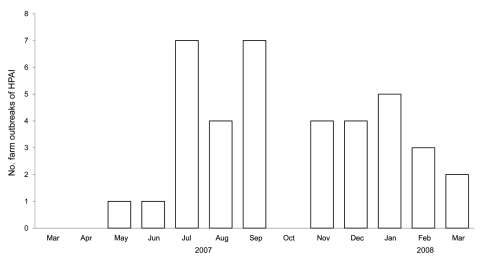
Number of highly pathogenic avian influenza (H5N1) outbreaks, by month, in 96 randomly selected small-holder duck farms, Central Java, Indonesia, March 2007–March 2008.

Of 96 study farms 34 (35%) across all 4 districts had HPAI outbreaks during the study period (Figure 3). One farm had 3 outbreaks and 2 farms had 2 outbreaks in different months; each of the remaining 31 farms had 1 outbreak. The numbers of outbreaks increased substantially from 1 each in May and June 2007 to 7 each in July and September 2007.

On 16 of the 34 farms that experienced HPAI outbreaks, combined swab samples were collected from 136 clinically normal birds (109 ducks, 27 chickens) at the same time as samples from dead birds on the same farm. For 11 (69%) of 16 outbreak farms, HPAI (H5) virus was isolated from 37 (27%) of 136 clinically normal birds (28 ducks, 9 chickens).

Carcasses of 180 marked and unmarked birds (59 ducks, 121 chickens) were obtained; HPAI (H5N1) virus was isolated from 65 (10 ducks, 55 chickens). Another 14 birds (3 ducks, 11 chickens) had most likely died from HPAI (H5) infection; we based this determination on 1) sudden death with or without clinical signs of HPAI (such as lethargy; swelling or discoloration of combs, wattles, and legs; nasal discharge; coughing and sneezing; diarrhea; and lack of coordination) and 2) isolation of HPAI (H5) virus from other dead birds within the flock at the same time. Therefore, a total of 79 (44%) of 180 birds most likely died from HPAI infection.

## Discussion

Scavenging duck farming has been proposed as an important contributor to HPAI in poultry flocks in Southeast Asia, predominantly on the basis of findings obtained through spatial analyses of national surveillance data of HPAI outbreaks (*23**–**25*). However, no field studies have investigated infection patterns over time in duck farming systems. Results of the current study indicate that scavenging ducks are a source of infection for other poultry and, possibly, for humans.

One explanation for the higher seroprevalence in ducks than in chickens is that HPAI (H5N1) virus circulated more successfully among ducks than among in-contact chickens; hence, ducks were more likely to harbor and transmit the virus. This could because of a higher risk for death among infected chickens, resulting in fewer surviving chickens with H5 antibodies, or to differences in scavenging behavior between ducks and chickens. Virulence of HPAI (H5N1) virus for ducks varies from inconsequential to highly lethal (*10**,**26*), and some of the 2003–04 Asian-lineage subtype H5N1 viruses can be shed by domestic ducks for up to 17 days postinfection (*11**–**12*). When viruses harbored by ducks are transmitted to gallinaceous species, such as chickens, severe clinical signs and high death rates can occur (*10*). However, for high-incidence HPAI outbreaks in chickens, virus excretion from infected ducks must be combined with an efficient reproductive number (R_0_) to produce secondary cases in a susceptible chicken population. R_0_ is influenced by the infectiousness of the agent causing the disease, the probability of transmission (determined by factors such as housing, mixing, and feeding practices), and the level of population immunity. During the 2004 HPAI (H5N1) epidemic in Thailand, R_0_ estimates were lower for backyard chickens than for broilers and layers (*27*). Birds of the latter 2 groups are typically housed together; but backyard chickens usually have less contact with each other. On our study farms, ducks usually grazed together, behavior conducive to virus circulation between ducks; individual in-contact chickens scavenged more independently. Another possible explanation for the difference in seroprevalences between poultry species is that duck flocks were exposed to HPAI more frequently than were chickens. Duck flocks may graze in the same rice fields where other potentially infected domestic or wild birds may have grazed.

The higher flock-level seroprevalence in ducks than in chickens was probably not biased substantially on differences in the numbers of birds sampled (10 ducks, 5 chickens) at each farm visit. In flocks where no study birds were detected with avian influenza (H5) virus or antibody, the virus may have been in other ducks or chickens in the same flock, and therefore our flock-level prevalence estimates underestimated the true flock prevalences. Because we sampled more ducks than chickens in each study flock, the risk for nondetection of infection was higher for chickens. However, on the basis of sample size calculations for assessing freedom from disease, this bias is unlikely to explain the differences in flock prevalences of antibodies between ducks and chickens (*28*). For example, if the true seroprevalence was 3% in populations of 100 ducks and 100 chickens, the probability of detecting at least 1 seropositive bird from 10 sampled ducks is 0.27 and, from 5 sampled chickens, 0.16. This equates to an OR of 1.9, which is substantially lower than the observed difference in flock-level prevalences in which the odds of duck flocks being seropositive were 12.4× higher than that of chicken flocks.

HI tests in which horse erythrocytes were used to detect avian influenza antibodies in human serum were more sensitive than HI tests in which chicken erythrocytes were used (*29*). Because OIE does not recommend the horse erythrocyte method for HI tests on poultry serum samples, it is rarely used in poultry diagnostics, although some evidence supports a higher sensitivity in these species (*30*). We compared HI tests based on horse and chicken erythrocytes by using serum samples from 60 ducks experimentally infected with 2 of the HPAI (H5N1) field isolates from this study. These tests showed substantial agreement when results were categorized as positive (>2^4^) or negative (κ = 0.74, 95% CI 0.57–0.90), although some samples tested with horse erthrocytes had higher titers than when tested with chicken erythrocytes. Thus, seroprevalence estimates may have been similar to those reported here had the HI tests been conducted by using horse erthrocytes. We suggest that OIE review this issue and, if warranted, modify the recommended diagnostic methods.

Virus shedding was reported in apparently clinically healthy birds on nearly 11/16 outbreak farms. Despite a high risk for death in chickens and some deaths in ducks, other birds carrying the virus appeared to be unaffected, which indicates that host-specific characteristics of susceptibility might have varied among birds. Alternatively, some of these virus-positive clinically normal birds could have been sampled early in infection and had not yet developed clinical signs. However, a small number of chickens in the longitudinal study had antibodies to avian influenza (H5), providing further evidence that some chickens survive infection. These birds could have been infected with low pathogenicity avian influenza (LPAI) viruses. To our knowledge, the prevalence of LPAI in poultry in Indonesia is unknown. However, influenza (H5N1) viruses isolated from dead and live birds in our study were confirmed to be highly pathogenic. Further molecular characterization of these isolates is under way (*31*).

The frequency of HPAI outbreaks varied throughout the study period. Outbreaks increased in July 2007 (the beginning of the dry season), coinciding with an increased proportion of flocks with seropositive ducks. This increase suggests that HPAI (H5) virus was circulating among more duck flocks during this time and may be related to the practice of herding free-ranging ducks to scavenge on paddy fields postharvest as described for Thailand and Vietnam (*24**–**25*). Intermingling of ducks on paddy fields may allow extensive opportunities for virus release and exposure and contact with wild birds that also feed on leftover rice (*25*). However, the relationship between rice farming and HPAI outbreaks in Indonesia is likely to vary from those in Thailand and Vietnam because of different climatic conditions and rice farming calendars. Rice farming in Central Java is less seasonal, and rice paddies are smaller, often not separated by wide waterways, unlike in the Mekong Delta of Vietnam. Other factors, such as the long distance movement of duck flocks, may influence outbreak patterns in Indonesia. Further studies are needed on the management and movement of duck flocks, HPAI transmission pathways between different poultry species, and the association between rice harvest activities and increased HPAI outbreaks
